# Supplementary material for: Thermal Stability and Purity of Graphene and Carbon Nanotubes: Key Parameters for Their Thermogravimetric Analysis (TGA)
Source: Nanomaterials (Basel). 2024 Oct 31;14(21):1754. doi: 10.3390/nano14211754 (PMC11547994; doi:10.3390/nano14211754)
Supplement: Supplementary file 1 [file nanomaterials-14-01754-s001.zip › nanomaterials-3272911-supplementary.pdf]

Thermal stability and purity of graphene and carbon nanotubes: key parameters for their thermogravimetric analysis (TGA)

Markus Martincic, Stefania Sandoval, Judith Oró-Solé, Gerard Tobías-Rossell*

Institut de Ciencia de Materiales de Barcelona (ICMAB-CSIC, Campus de la UAB, 08193 Bellaterra, Barcelona, Spain

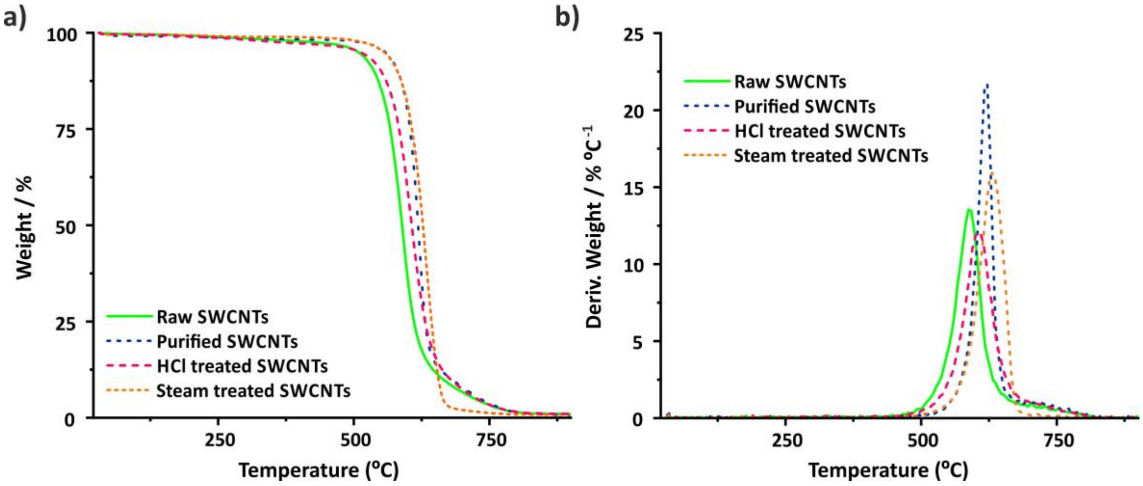

Figure S1. a) TGA and **b)** DTG curves of raw and post-synthesis treated SWCNTs performed on a Netzsch instrument, model STA 449 F1 Jupiter® (Selb, Germany). The samples were analyzed under flowing air at a 10 °C min^{-1}, up to 900 °C. Purified SWCNTs refers to a sample of raw/pristine SWCNTs treated with steam and HCl.

Table S1. Temperature events that occur during the oxidation of SWCNTs.

Sample	T_{onset}	T_0	T_{50}	T_{offset}	%Fe
Raw SWCNTs	557	589	589	666	0.71%
Purified SWCNTs (steam + HCl)	598	619	619	773	0.48%
HCl treated SWCNTs	573	607	607	643	0.66%
Steam treated SWCNTs	599	632	627	656	0.55%

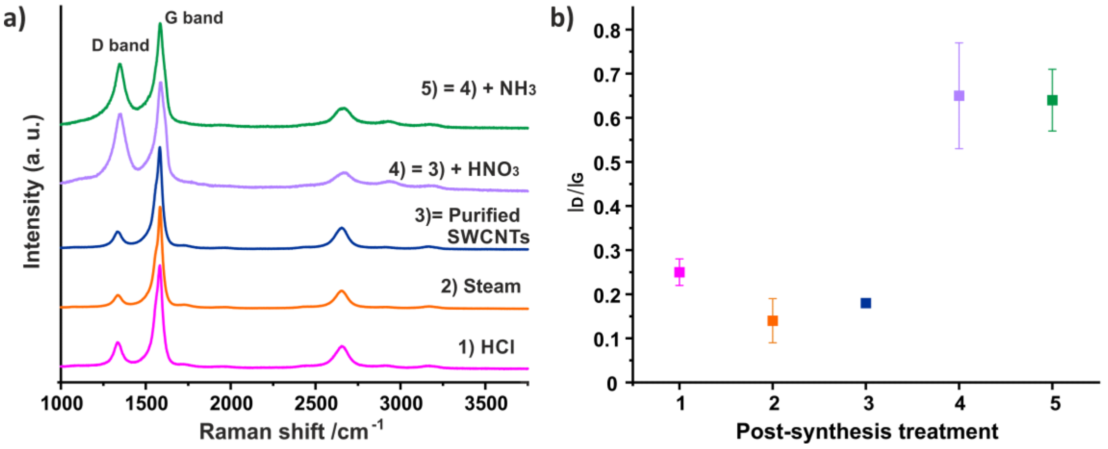

Figure S2. a) Raman spectra and **b)** I_D/I_G ratios obtained from the corresponding Raman spectra of post synthesis treated SWCNTs, obtained using a 532 nm laser. Purified SWCNTs **3)** refers to a sample of raw/pristine SWCNTs treated with steam and HCl.

Table S2. Position and intensity ratios of both D and G bands determined from Raman spectra of post-synthesis treated SWCNTs.

Sample	D peak	G peak	I_D/I_G
HCl treated SWCNTs, **1)**	1335.5	1579.7	25 ± 3
Steam treated SWCNTs, **2)**	1333	1577	14 ± 5
Purified SWCNTs: steam+ HCl, **3)**	1335.5	1581	18 ± 0.3
O-functionalized SWCNTs, **4)**	1346.7	1583	65 ± 12
N-doped SWCNTs, **5)**	1346.7	1585	64 ± 7

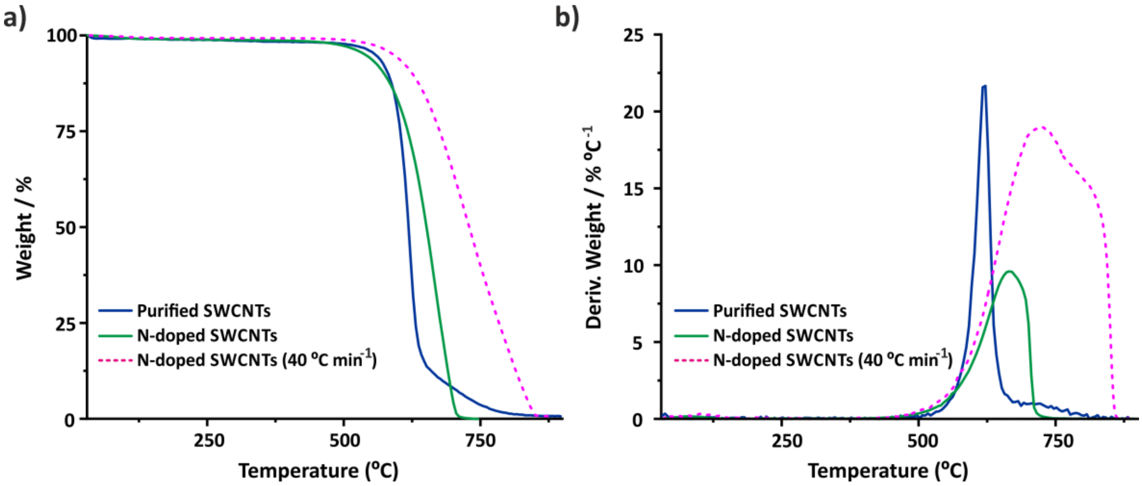

Figure S3. a) TGA and **b)** DTG curves of purified (continuous blue line) and N-doped SWCNTs (continuous green line) analyzed under flowing air at 10 °C min^{-1} and N-doped SWCNTs (dotted magenta line), analyzed under flowing air at 40 °C min^{-1} . TGA were performed on a Netzsch instrument, model STA 449 F1 Jupiter® (Selb, Germany).

Table S3. Temperature events that occur during the oxidation of N-doped carbon nanomaterials.

**Heating rate (N-doped SWCNTs)**	T_{onset}	T_0	T_{50}	T_{offset}
10 ºC min^{-1}	608	567	652	702
40 ºC min^{-1}	632	725	730	833
